# Evaluation of the Properties of Bioactive Mesoporous Glasses Doped with Cerium and Loaded with Polyphenols

**DOI:** 10.3390/ma18030709

**Published:** 2025-02-06

**Authors:** Alessia Giordana, Chiara Cavazzoli, Francesca Fraulini, Paolo Zardi, Alfonso Zambon, Giuseppina Cerrato, Gigliola Lusvardi

**Affiliations:** 1 Dipartimento di Chimica, Università degli Studi di Torino, 10125 Torino, Italy; alessia.giordana@unito.it; 2 Dipartimento di Scienze Chimiche e Geologiche, Università degli Studi di Modena e Reggio Emilia, 41125 Modena, Italy; chiara.cavazzoli@unimore.it (C.C.); francesca.fraulini@unimore.it (F.F.); paolo.zardi@unimore.it (P.Z.); alfonso.zambon@unimore.it (A.Z.)

**Keywords:** bioactive glasses, cerium, biomolecules, antioxidant activities

## Abstract

(1) Background: The onset of inflammation and oxidative stress after biomaterial implantation can lead to complications and prolonged recovery times. To address this, bioactive mesoporous glasses doped with cerium (0, 3.6 and 5.3 mol%) were loaded with three different biomolecules—3-hydroxyflavone, quercetin and morin hydrate—to enhance antioxidant properties while preserving bioactivity. (2) Methods: Elemental analysis, specific surface area determination, spectroscopic techniques, evaluation of antioxidant activity and in vitro bioactivity assessment were performed to characterize mesoporous glass loaded with biomolecules. (3) Results: Biomolecule loading gives values in the range of 0.5–2.0% and 10.3–39.6% for loading content and loading efficiency, respectively. The loading order is quercetin > morine hydrate > 3-hydroxyflavone, and a cerium percentage of 3.6 seems to be a good compromise. The antioxidant properties evaluated on both solids and solutions in contact with simulated biological fluids improve markedly over loaded glasses, and the most promising results are obtained with quercetin. In the most efficient systems, the bioactivity results were delayed and more evident at longer times (168 h) but were still retained. (4) Conclusions: We obtained new materials still bioactive with improved antioxidant properties that can be proposed for the regeneration of both hard and soft tissues.

## 1. Introduction

Bioactive glasses (BGs) are widely utilized in medicine as bone fillers, scaffolds, and implant coatings due to their remarkable capacity to promote bone regeneration. Since the introduction of 45S5 Bioglass^®^ [1], their applications have broadened significantly, paving the way for numerous biomedical innovations. The versatility of BGs lies in their ability to adapt across various forms, such as powders, coatings, 3D scaffolds, and fibers, and in the incorporation of therapeutic inorganic ions (TIIs), which enhance key properties including osteogenesis, angiogenesis, antibacterial activity, and cementogenesis [2]. The insertion of a biomaterial through surgery often results in the onset of local inflammation and is linked to the production of reactive oxygen species (ROS), thus creating a condition of oxidative stress, which in turn increases inflammation, leading to further ROS generation [3,4]. The ability to convert ROS into non-hazardous species is therefore a desirable feature of a biomaterial in order to reduce post-implantation complications and the need for lengthy drug treatments and recovery times [5]. A suitable strategy to this end is adding antioxidant properties to biomaterial by doping with TIIs and loading with biomolecules and/or drugs [6,7]. BGs are particularly suited to this purpose due to their surface reactivity. Among TIIs, cerium, whose compounds are known for therapeutic applications, is of particular interest as an antioxidant [8,9], as it is able to inhibit ROS production and regulate their levels within the microenvironment, showing antioxidant and anti-inflammatory activities [10,11,12,13]. Moreover, cerium-doped BGs (BGsCe) are produced by different synthetic methods, each of which corresponds to a specific category: melted quenched glasses (MQGs), sol–gel bioactive glasses (SGGs) and mesoporous bioactive glasses (MBGs). The reactivity of BGs depends on the method of synthesis; MBGs are more reactive due to their high pore volume and specific surface area (SSA) [9,14]. Furthermore, MBGs are effective drug delivery systems (DDSs), allowing for high loading efficiency combined with slow and controlled release kinetics [15,16,17,18]. Recently, we developed a range of BGsCe that differ in synthesis, composition, reactivity and in vitro properties [19,20,21]. We demonstrated that the addition of cerium does not significantly alter the bioactivity and the antibacterial properties of the biomaterial, while it has a positive effect on biocompatibility and improves antioxidant properties [9,11,13].

The use of natural polyphenols for health has increased considerably in recent years [11,22,23] due to their antioxidant, anti-inflammatory, antibacterial, osteoinductive and anticancer properties. The activity of polyphenols does not depend solely on the concentration of these biomolecules in food; it also depends on their bioavailability following absorption, metabolization and tissue distribution. Unfortunately, the exposures required to achieve a therapeutic effect by polyphenols are often not achievable in vivo solely through diet. Loading a biomaterial with polyphenols is therefore a strategy to optimize a controlled release of these biomolecules, to increase their bioavailability and to achieve synergistic effects between the properties of the biomaterial and biomolecules [11,23,24,25].

In previous works [11,23], we reported a further improvement in the antioxidant properties of cerium-doped MBGs (MBGsCe) loaded with a mixture of polyphenols extracted from chestnuts (POLY) and also confirmed the ability of the loaded MBGs to retain bioactivity in a biological medium.

**Figure 1:**
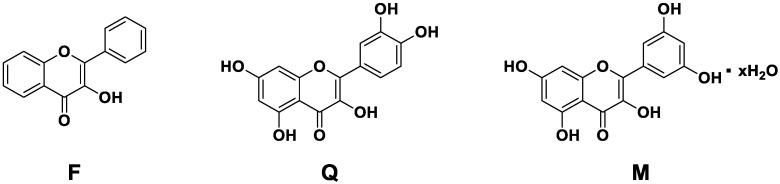
Chemical structure of 3-hydroxyflavone (F), quercetin (Q) and morin hydrate (M).

The aim of the present study is to evaluate other biomolecules in the same setting in order to identify hybrid materials with optimal antioxidant properties and controlled drug delivery behavior. We report therefore a multiparametric evaluation of MBGsCe loaded with three different biomolecules: 3-hydroxyflavone, quercetin and morin hydrate, whose structure is reported in Figure 1.

While not found in nature, 3-hydroxyflavone represents the basic backbone of flavonoids, and it is investigated for its various pharmacological properties [26], such as antiviral, anti-inflammatory and antioxidant. Furthermore, the strong electron conjugation of the aromatic systems also confers on 3-hydroxyflavone a fluorescent property, which makes it usable as an imaging agent for therapeutic purposes [26]. Distinctly from quercetin and morine, 3-hydroxyflavone does not present catechol groups, which are commonly found in natural polyphenols [27].

Quercetin is a flavonoid found in fruit and vegetables, and it has been indicated for treating various oral diseases, including oral cancer, periodontitis, caries, recurrent aphthous ulcers, endodontic root canal and dental restoration [28].

Morin hydrate is a bioflavonoid obtained mainly from the fruits, stems and leaves of plants in the *Moraceae* family, and it has been indicated for various life-threatening chronic and degenerative diseases [29].

In this study, we present the evaluation of the loading extent of these biomolecules on MBGsCe, which is followed by the study of their stability, antioxidant properties and bioactivity upon loading.

## 2. Materials and Methods

### 2.1. MBGsCe Preparation and Biomolecules’ Loading

Three MBGsCe (Table 1) containing different cerium amounts (0, 3.6 and 5.3 mol%) were synthesized by a sol–gel EISA modified method, using TEOS, TEP, Ca(NO_3_)_2_ and Ce(NO_3_)_3_ as precursors, as reported in our previous papers [9,10,11,13,30] sieved to a 212–355 µm dimension and loaded with 1.0 mg/mL of the biomolecules’ loading solutions. Specifically, in the EISA method, we used the Pluronic P123 as a self-assembling polymer. The procedure is the following: first, 4.5 g of the surfactant Pluronic P123 (Sigma-Aldrich, Merck Life Science S.r.l. Milano, Italy, M_n_~5800) was dissolved by magnetic stirring (~1 h) in 85 mL of ethanol (C_2_H_5_OH, Sigma-Aldrich, Merck Life Science S.r.l. Milano, Italy, ≥99.8) containing 1.2 mL of a 10% HCl (Sigma Aldrich, Merck Life Science S.r.l. Milano, Italy) solution; then, the appropriate amounts of TEOS (Sigma-Aldrich, Merck Life Science S.r.l. Milano, Italy, ≥99%), TEP (Sigma-Aldrich, Merck Life Science S.r.l. Milano, Italy, Italia, ≥99.8%), Ca(NO_3_)_2_·4H_2_O (Sigma-Aldrich, Merck Life Science S.r.l. Milano, Italy, ≥99%) and Ce(NO_3_)_3_·6H_2_O (Sigma-Aldrich, Merck Life Science S.r.l. Milano, Italy, 99%) were added under continuous stirring in 3 h intervals at room temperature.

The concentrations of the loading solutions derive from the optimal values reported in our previous manuscripts [11,23].

3-Hydroxyflavone, quercetin and morin are 98, 95 and 100% pure, respectively, and from Sigma Aldrich. FT-IR spectra and XRPD pattens of biomolecules are reported in Supplementary Materials Figures S1 and S2). Then, 1.0 mg/mL loading solutions were prepared by dissolving the biomolecules in ethanol for 2 h under magnetic stirring. The loading was carried out by soaking 0.1 g of each sample for 3 h at 37 °C in 5 mL of the biomolecules’ loading solution. All the samples were covered with aluminum foils to prevent exposure to light.

The name of the samples obtained and studied is indicated as “MBGsCepoly” with Ce = 0, 3.6, 5.3 mol% poly = F, Q, M for 3-hydroxyflavone, quercetin and morin, respectively. The use in figure captions of the term UL indicates that the samples are not loaded.

### 2.2. Surface Activation

The surface activation of each sample was carried out according to the literature [32,33,34] in order to free the hydroxyl groups on the surface and promote loading. First, 0.4 g of each sample was suspended in 5 mL of acetone and washed for 5 min in an ultrasonic bath; then, it was rinsed three times with 5 mL of double-distilled water, under sonication, and finally soaked in the loading solution.

### 2.3. Elemental Analysis (EA)

Elemental analysis (EA) was carried out with a FLASH 2000 (Thermo Fisher Scientific Inc., Waltham, MA, USA) analyzer in order to quantify the amount of biomolecules in the loaded MBGs by measuring C (%). The results for each biomolecule will be expressed as loading content, LC (%) and loading efficiency LE (%) calculated as follows, where m = mass.

These data derive from three replicated experiments.

$$LC\left(\%\right)=\frac{MM\left(biomolecule\right)}{mol\left(Cintobiomolecule\right)\times MA\left(C\right)}\times C\left(\%\right)$$

$$LE\left(\%\right)=\frac{m_{\left(biomoleculeloaded\right)}}{m\left(biomoleculeloadingsolution\right)}\times 100$$

### 2.4. Folin–Ciocalteu (FC) Method

A modified Folin–Ciocalteu (FC) method was applied to quantify the amount of loaded biomolecules via UV-Vis determination (JASCO V-570, Mettler Toledo, Columbus, OH, USA). First, 300 µL of biomolecules’ solution after loading was mixed with 900 µL of double-distilled water, 75 µL of Folin–Ciocalteu reagent (Sigma Aldrich) and 225 µL of Na_2_CO_3_ 20% (*w*/*v*). A calibration curve for each biomolecule was also prepared. After 2 h, UV-Vis measurement was carried out (λ = 754 nm).

The results are expressed as LC (%) and LE (%), which were calculated as follows:

$$LE\left(\%\right)=\frac{C_{i}-C_{f}}{C_{i}}\times 100$$

$$LC\left(\%\right)*=\frac{LE\left(\%\right)\times C_{i}\times V_{i}}{m\left(MBG\right)}\times 100$$

* Derived from LE (%). Where C_i_ = biomolecules’ loading solution concentration (mg/mL) before loading; C_f_ = biomolecules’ loading solution concentration (mg/mL) after loading; V_i_ = biomolecules’ loading solution volume (mL); m (MBG) = MBGs mass (mg).

Due to the nature of the biomolecule, it was not possible to perform this test on the MBGsCe loaded with 3-hydroxyflavone.

### 2.5. FT-IR Spectroscopy

FT-ATR (Attenuated Total Reflection) spectra of MBGsCe and MBGsCepoly were recorded with a VERTEX 70 spectrophotometer equipped with a Harrick MVP2 ATR cell and a DTGS detector (64 scans, 4 cm^−1^ resolution) (Bruker, Karlsruhe, Germany). FT-IR spectra were recorded with same instrument as for the FT-ATR ones: samples were prepared in the form of self-supporting pellets (ca. 10 mg cm^−2^) to obtain a sufficiently thin pellet to be inspected in transmission mode. However, this condition should have enough accuracy regarding all the spectral components that we need to characterize our materials, in particular in the region below 1400 cm^−1^.

### 2.6. Specific Surface Area (SSA) Determination

Specific surface area (SSA) was evaluated before and after loading in order to assess the possible textural changes arising from this process. SSA was determined by nitrogen adsorption porosimetry using a Chemisorb 2750 (Micromeritics SRL, Milano, Italy) and the Brunauer–Emmett–Teller (BET) method [35].

### 2.7. Antioxidant Activity Assays

The antioxidant properties of MBGsCepoly were evaluated by two different methods described below. The first is named superoxide dismutase (SOD)-like activity [11,23,36] in analogy with the role of the SOD enzyme. The second is the 1,1-diphenyl-2-picrylhydrazyl (DPPH) removal assay, which is a bioanalytical method used to measure radical scavenging activity (RSA) [37,38,39].

#### SOD-like Activity

SOD-like activity tests were performed using the SOD Determination Kit (Sigma Aldrich) adapted for a UV-Vis spectrophotometer (JASCO V-570, Mettler Toledo, Columbus, OH, USA). The test was performed on MBGsCepoly powders and on Dulbecco’s phosphate-buffered saline, D8537 (DPBS) solution after 1, 4, 24, and 48 h of soaking with the powders (MBGsCepoly (mg)/DPBS (mL) = 75/50). The SOD-like activity is expressed as the rate of inhibition (I.R.%) of the formation of a water-soluble formazan dye formed upon the reduction of a tetrazolium salt (WST-1) by the superoxide anion, which was catalyzed by xanthine oxidase (XO) and inhibited by SOD. Values are reported as means ± standard deviation from three replicated experiments. A *t*-test was used to compare the means, and statistically significant differences were defined as those with a *p*-values less than 0.01.

### 2.8. RSA

The antioxidant capacity was evaluated using the DPPH test for a UV-Vis Spectrophotometer Cary3500 Compact UV-Vis (Agilent, Santa Clara, CA, USA), monitoring the variation in absorbance at 524 nm. The test was performed after 1, 4, 24, and 48 h of DPBS soaking with the powders (MBGsCepoly (mg)/DPBS (mL) = ratio of 9.0/6.0). The test was performed in triplicate and the solutions were merged prior to the analysis. Then, 1.2 mL of DPPH ethanolic solution (5 × 10^−5^ M) was added to 1.2 mL of DPBS soaking solution, and the absorbance was monitored for 45 min, acquiring spectra every three minutes.

The following formula was used to calculate the percentage of RSA:

$$\%\;\mathrm{RSA}=\frac{A_{0}-A_{30}}{A_{0}}\times 100$$

where A_0_ = absorbance at time zero; A_30_ = absorbance after 30 min.

### 2.9. In Vitro Bioactivity Assessment

MBGsCepoly were soaked in a simulated biological fluid (SBF) prepared as described by Kokubo [40,41] at 37 °C for 24, 72 and 168 h in order to verify the retention of bioactivity (formation of an apatitic layer constituted of hydroxyapatite, Ca_10_(PO_4_)_6_(OH)_2_, HA) by the loaded materials [40,41,42]. After soaking, FT-ATR spectra were collected on a Perkin Elmer 1600 spectrometer (Perkin Elmer, Waltham, MA, USA) in the range of 400–4000 cm^−1^, 180 scans to identify the characteristic bands of HA. Moreover, mineralogical evaluations (by X-ray Powder Diffraction, XRPD) were also carried out using an X’Pert PRO diffractometer (Malvern PANanalytical, Almelo, The Netherlands) (Cu Kα radiation, 5–70°, 2θ range) to detect the characteristic peaks of HA. Scanning Electron Microscopy (SEM) with a JSM-6010LA microscope (JEOL, Tokyo, Japan) equipped with electron-dispersive spectroscopy, EDS, Leica Microsystems, Wetzlar, Germany) was used to evaluate morphological changes on the surface.

## 3. Results and Discussion

### 3.1. Loading Evaluation

#### EA and BET

LC (%) and LE (%) are reported in Table 2; these results indicate an efficient loading of the selected biomolecules on MBGsCe with values ranging from 0.5 to 2.0% and from 10.3 to 39.6% for LC and LE, respectively. The presence of cerium lowers these values without markedly compromising the extent of loading.

**Figure 2:**
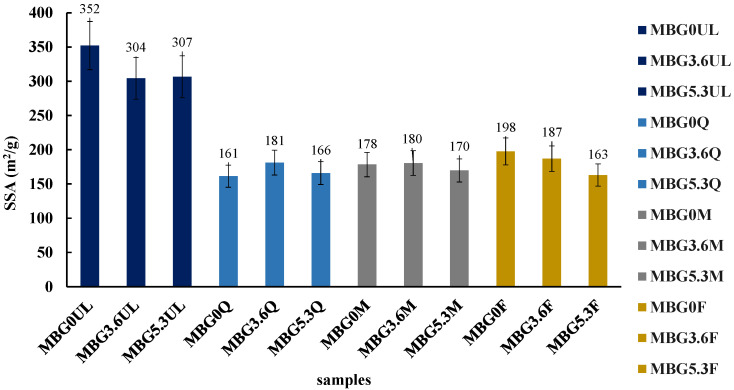
SSA (m^2^/g) of MBGsCe before and after biomolecules loading. Tests were performed in triplicate. Data are presented as averages with error bars representing the SD.

The loading effect was also assessed by measuring the specific surface area (SSA), as reported in Figure 2.

The unloaded MBGsCe possess high SSA (300–350 m^2^/g) consistently with their mesoporous structure and regardless of cerium content; loaded MBGsCe showed a significant decrease in SSA (160–200 m^2^/g), likely due to pore occlusion, which does not depend on the type of loaded molecules.

### 3.2. FT-IR

**Figure 3:**
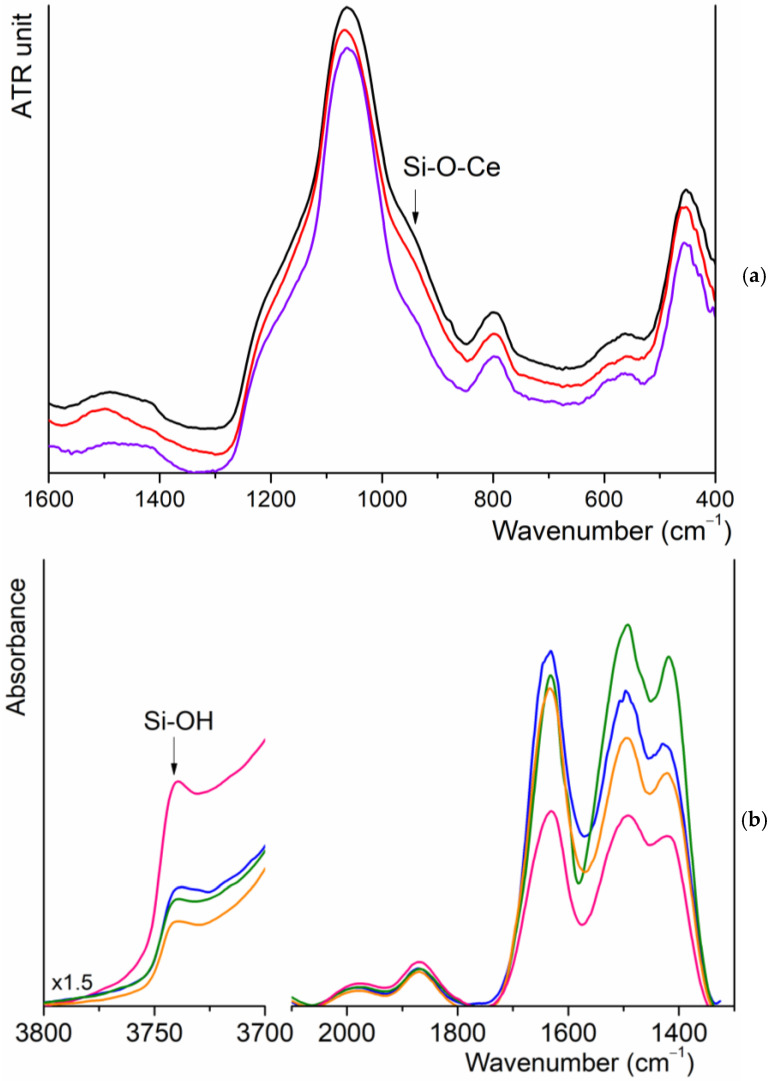
(**a**) ATR spectra of MBG0 (purple), MBG3.6 (red) and MBG5.3 (black); (**b**) FT-IR spectra of MBGs3.6: UL (pink), Q (blue), M (orange) and F (green).

In the ATR spectra of samples, the characteristic bands of silica can be observed; in particular, the asymmetric stretching mode of O-Si-O units generates an intense band in the spectral range of 1300–900 cm^−1^ (Figure 3a). In the presence of cerium, there is a shoulder at 940 cm^−1^ that can be related to Si-O-Ce modes [43].

The spectra of MBGsCepoly samples are very similar, and no signal attributable to biomolecules is identifiable, probably for the low quantity loaded on each sample. Trying to improve the intensity of the signals, we recorded the FT-IR spectra of a pure self-supported pellet. For both unloaded and loaded MBG3.6 samples, see Figure 3b, it is possible to recognize the overtone of silica (2000–1800 cm^−1^), the bending mode of absorbed water molecules at 1630 cm^−1^ and a multiple band with maxima at 1450 and 1410 cm^−1^, which are, respectively, attributable to the formation of carbonate groups on the surface of the materials. Comparing the normalized spectra, there is an increase in the intensity of the former band due to the superimposition with the most intense modes of polyphenols molecules. At higher wavenumbers, the signal of isolated silanol surface groups at 3740 cm^−1^ is well recognizable, whose intensity decreases in MBGsCepoly, suggesting that polyphenols adsorption involved the formation of H bonding with these groups. Similar results were observed for higher cerium amounts.

### 3.3. Antioxidant Properties

**Figure 4:**
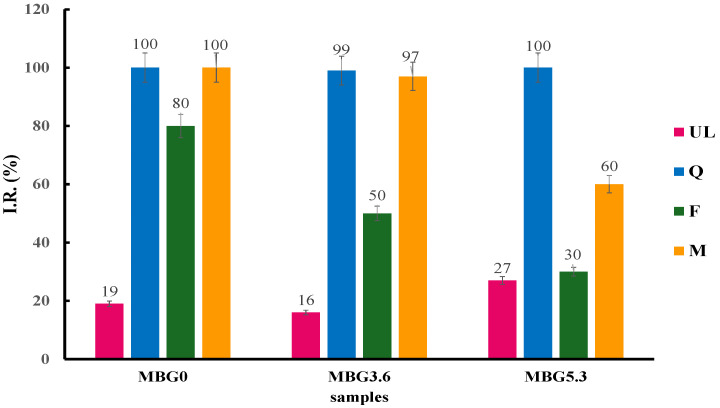
SOD-like activity of MBGsCepoly. Tests were performed in triplicate. Data are presented as averages with error bars representing the SD.

The results of the SOD assay on the MBGsCepoly (Figure 4) indicate that loading with biomolecules causes a significant improvement in the SOD-like activity of the hybrid materials, especially with Q-loaded samples, that reach full I.R. at all cerium contents. Interestingly, the SOD-like activity of F-loaded samples decreases significantly at higher amounts of cerium: 80, 50 and 30 I.R. (%) for MBG0, MBG3.6 and MBG5.3, respectively. M-loaded samples show intermediate behavior with only the MBG5.3 sample showing a marked decrease with I.R. at 60%.

**Figure 5:**
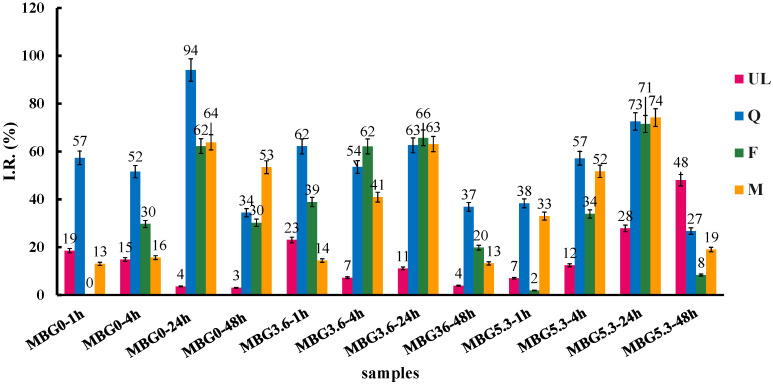
SOD-like activity of MBGsCepoly after DPBS soaking. Tests were performed in triplicate. Data are presented as averages with error bars representing the SD.

We then tested the SOD-like activity the solution after DPBS soaking (Figure 5).

All the eluates show a growing SOD-like activity up to 24 h; at 24 h, all samples have I.R. around 60–70% with the single exception of MBG0Q that show I.R > 90%. At 48 h, all samples show a marked decrease in SOD-like activity, except for unloaded MBG5.3, which presents a marked activity at longer times of release. This latter result suggests a limited radical scavenging activity linked to the dissolved cerium ions, as we observed in previous studies [11].

**Figure 6:**
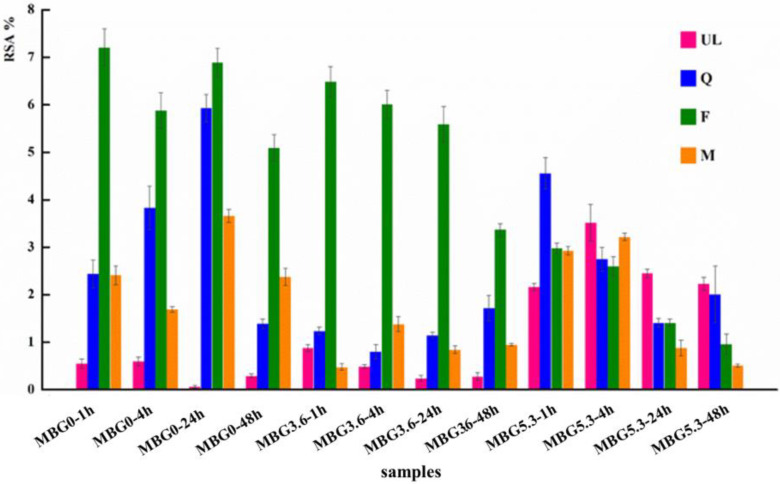
RSA values of MBGsCepoly after DPBS soaking. Tests were performed in triplicate. Data are presented as averages with error bars representing the SD.

To further profile this behavior, we decided to investigate the antioxidant activity of the eluates by the DPPH test to measure their RSA [37,38,39]. In agreement with what was observed with the SOD assay, the results (Figure 6) indicate an improvement in the antioxidant activity with respect to unloaded MBGs, at least for MBG0 and MBG3.6, and increasing RSA at longer soaking times up to 24 h.

While in the SOD assay all loaded samples behaved in a roughly similar manner, the F-loaded samples present higher RSA than Q and M at all time points for MBG0 and MBG3.6 with a moderate decrease in RSA only a later time points. Albeit flavonoids with higher RSA values usually present a larger number of hydroxyl groups in their structures [44]; discrepancies between the results of different antioxidant assays are commonly observed [45,46]. Given the absence of catechol groups in F, the difference between the results in the two assays could be linked to a different affinity of the polyphenols with the molecular probes of the two assays, namely the small, charged superoxide anion and radical DPPH. The trend in RSA for MBGs5.3 is again comparable with that of the SOD-like assay with unloaded MBG5.3 showing a detectable antioxidant activity and a decrease in the RSA of the functionalized MBGs5.3 at longer time points. As observed for the SOD assay at 48 h, the RSA of all loaded samples with polyphenols decreases with the specific exception of MBG0F. The general decrease in RSA at longer time points again suggests a partial degradation of the polyphenols after prolonged time in solution.

Indeed, the quantification of the released F, M and Q in DPBS was complicated by the fact that the UV-Vis spectra of release solutions do not correspond to that of the starting molecule, confirming the partial degradation of the polyphenols in the tested solution.

### 3.4. In Vitro Bioactivity Assessment

From FT-IR measurements, the presence of the most important characteristic bands of HA is discussed, and the results are shown in Table 3. The results indicate that biomolecule loading delays but does not inhibit crystalline HA formation, which is in agreement with previous studies that indicated at least 168 h as the time required for HA [11,23] identification. The behavior of biomolecules is related to the presence of cerium, which is in agreement as widely discussed with increased structural cross-linking. Specifically, from a qualitative point of view, F best preserves the bioactivity for all MBGsCe studied (Figure S3).

**Figure 7:**
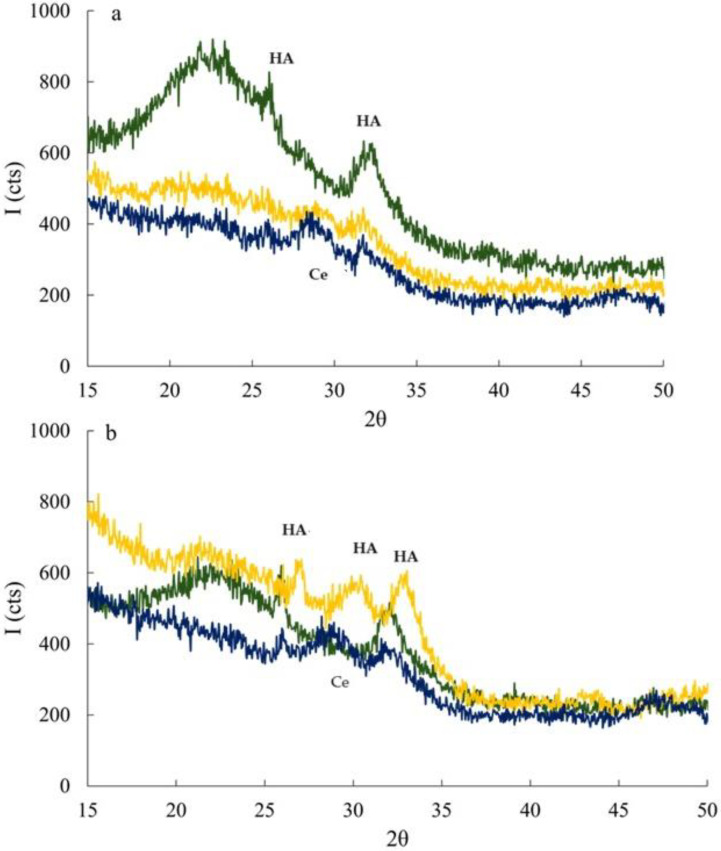
XRPD patterns of MBG0 (green), MBG3.6 (yellow) and MBG5.3 (blue) loaded with F after 72 (**a**) and 168 (**b**) h of SBF soaking. HA = Ca_10_(PO_4_)_6_(OH)_2_, Ce = CePO_4_.

XRPD patterns of MBGsCe loaded with Q, M were not reported, as the formed apatitic phase proved to be highly amorphous, making it poorly visible. Regarding the loading with F, with and without cerium, after 72 and 168 h in SBF (Figure 7a,b), some of the characteristic peaks of HA, at 26 and 32, 33 (°2*θ*), PDF [47] are recognized. The presence of a competitive phosphatic phase, CePO_4_ PDF [47], is also observed, with peaks at ~29 (°2*θ*); this is consistent with the reported mechanism in the literature, as it is a compound with a solubility product conducive to precipitation [11].

**Figure 8:**
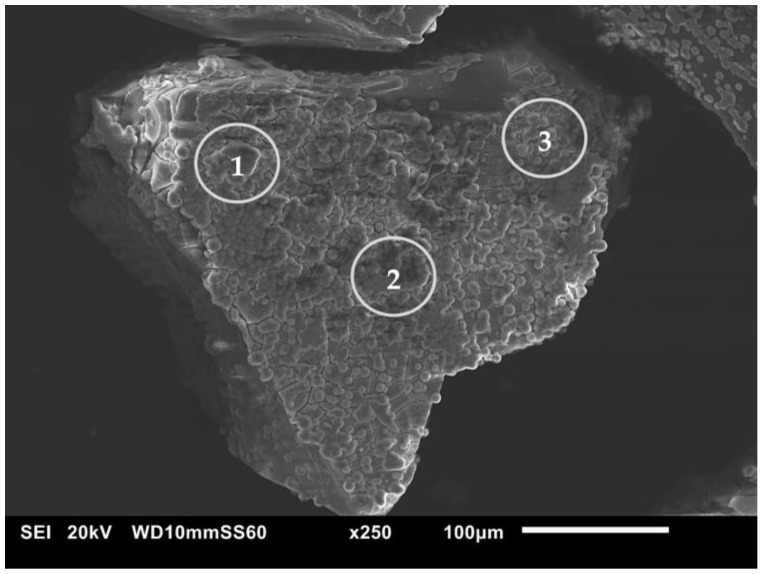
SEM micrograph of MBG3.6F after 168 h of SBF soaking.

The presence of HA was also verified for the most representative sample, MBG3.6F, via SEM-EDS analyses. A micrograph (Figure 8) reveals the reacted surface (circled in gray), and it is possible to observe the formation of more clear aggregates of spherical shape. Moreover, a significant increase in the CaO and P_2_O_5_ oxides was observed when comparing the values obtained from the EDS analysis (Table 4).

## 4. Conclusions

The objective of this study was to assess the degree of loading of three biomolecules, 3-hydroxyflavone (F), quercetin (Q), and morin hydrate (M), onto MBGsCe, resulting in the evaluation of their stability, antioxidant properties and bioactivity upon loading.

The loading of the studied biomolecules was successful with values in the range of 0.5–2.0% and 10.3–39.6% for loading content and loading efficiency, respectively.

The presence of cerium can lower loading values, but a cerium concentration of 3.6 is a good compromise, and among the various biomolecules studied, the best loading order is as follows: MBG3.6Q > MBG3.6M > MBG3.6F

The SSA decreased generally from about 300 to 200 m^2^/g in accordance with partial pore occlusion, which, however, does not compromise the mesoporous structure of the studied glasses, and also cerium does not markedly affect the SSA values.

Antioxidant properties increase compared to unloaded glasses depend on the type of biomolecule and agree with the loading values trend. In all cases, after 24 h, antioxidant activity tends to be depleted.

Finally, the bioactivity is retained at longer times (168 h) for MBGsCe that have better antioxidant properties.

The combination of new antioxidant properties with persistent bioactivity allows us to state that these new materials may be proposed to propose new controlled biomolecules’ delivery systems for both hard and soft tissues regeneration.

## Figures and Tables

**Table 1:** Nominal composition (mol%) of the MBGsCe [31].

MBGsCe	SiO_2_	CaO	P_2_O_5_	CeO_2_
MBG0	80	15	5	-
MBG3.6	77.1	14.5	4.8	3.6
MBG5.3	75.8	14.2	4.7	5.3

**Table 2:** LC (%) and LE (%) of MBGsCepoly calculated by EA.

MBGsCepoly	LC (%)	LE (%)
MBG0Q	2.0	39.6
MBG3.6Q	1.2	23.2
MBG5.3Q	1.0	19.5
MBG0M	1.1	21.7
MBG3.6M	0.5	10.3
MBG5.3M	0.6	11.7
MBG0F	1.7	34.9
MBG3.6F	1.0	20.1
MBG5.3F	0.6	11.9

Similar results were obtained with the FC method and are shown in Supplementary Materials (Table S1).

**Table 3:** FT-IR characteristic bands of HA after 24 (gray), 72 (blue) and 168 (green) h.

MBGsCe	Wavenumber (cm^−1^)					
	605	565	605	565	605	565
MBG0	+	+	+++	+++	+++	+++
MBG3.6	+	+	++	++	+++	+++
MBG5.3	+	+	++	++	+++	+++
MBG0Q	+	+	+	+	+	+
MBG3.6Q	−	−	−	−	+	+
MBG5.3Q	−	−	−	−	+	+
MBG0F	+++	+++	+++	+++	+++	+++
MBG3.6F	−	−	+	+	+++	+++
MBG5.3F	−	−	++	++	+++	+++
MBG0M	+++	+++	+++	+++	+++	+++
MBG3.6M	−	−	−	−	+	+
MBG5.3M	−	−	−	−	+	+

(+ present, ++ intense, +++ very intense band, − absent).

**Table 4:** EDS oxides compositions (mol %) of MBGs3.6F before and after 168 h of SBF soaking.

MBG3.6F	SiO_2_	P_2_O_5_	CaO	CeO_2_
before	77.1	4.8	14.5	3.6
after (circle 1)	2.6	23.6	73.8	-
after (circle 2)	3.9	20.7	75.4	-
after (circle 3)	3.3	23.3	73.4	-

## Data Availability

The original contributions presented in this study are included in the article/Supplementary Material. Further inquiries can be directed to the corresponding authors.

## Supplementary Materials

The following supporting information can be downloaded at: https://www.mdpi.com/article/10.3390/ma18030709/s1, Figure S1: FT-IR spectra of F (a), Q (b), and M (c); Figure S2: XRPD patterns of F (a), Q (b), and M (c); Figure S3: FT-IR spectra of MBG3.6 and MBG5.3 loaded with F after 72 (blue-red) and 168 (orange–light blue) in SBF; Table S1: LC (%) and LE (%), calculated for MBGsCepoly with FC method.
