# Supplementary material for: Evaluation of the Properties of Bioactive Mesoporous Glasses Doped with Cerium and Loaded with Polyphenols
Source: Materials (Basel). 2025 Feb 6;18(3):709. doi: 10.3390/ma18030709 (PMC11821127; doi:10.3390/ma18030709)
Supplement: Supplementary file 1 [file materials-18-00709-s001.zip › materials-3436904-supplementary.pdf]

materials

MDPI

Supplementary Information (SI)

Table S1. LC (%) and LE (%), calculated for MBGsCepoly with FC method.

MBGsCepoly	LC (%)	LE (%)
MBGQ	1.6	26.9
MBG3.6Q	1.5	26.5
MBG5.3Q	2.4	41.1
MBGM	0.8	15.4
MBG3.6M	0.7	13.1
MBG5.3M	1.0	19.5

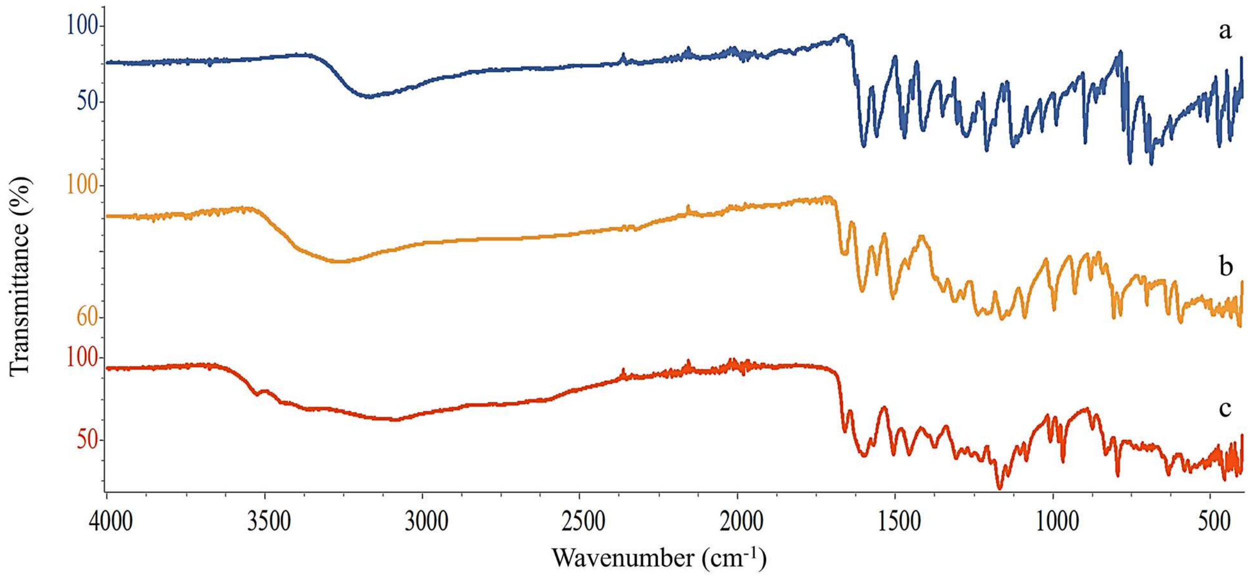

Figure S1 FT-IR spectra of F(a), Q(b), and M(c)

 https://doi.org/10.3390/ma18030709

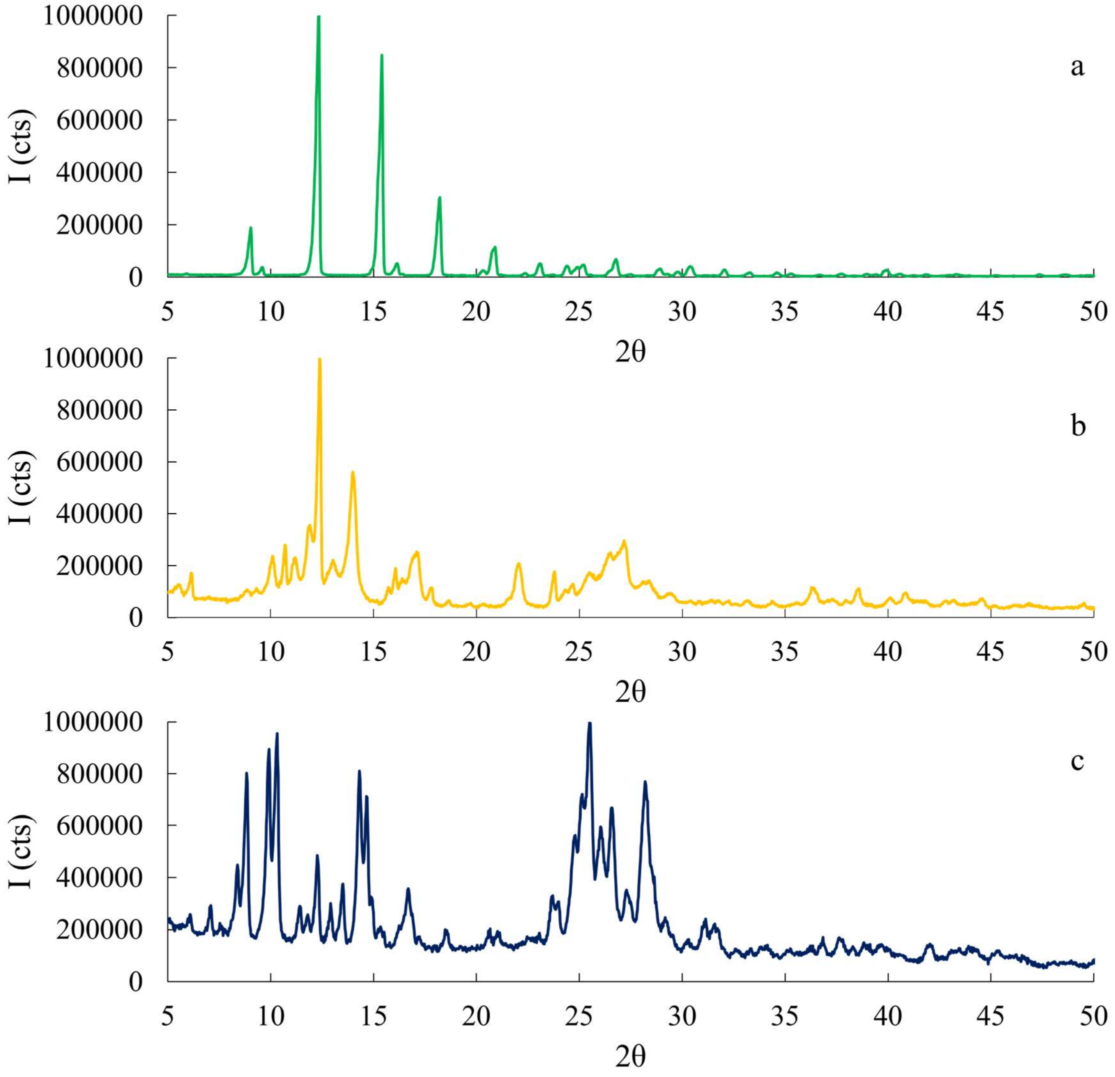

Figure S2 XRPD patterns of F(a), Q(b), and M(c)

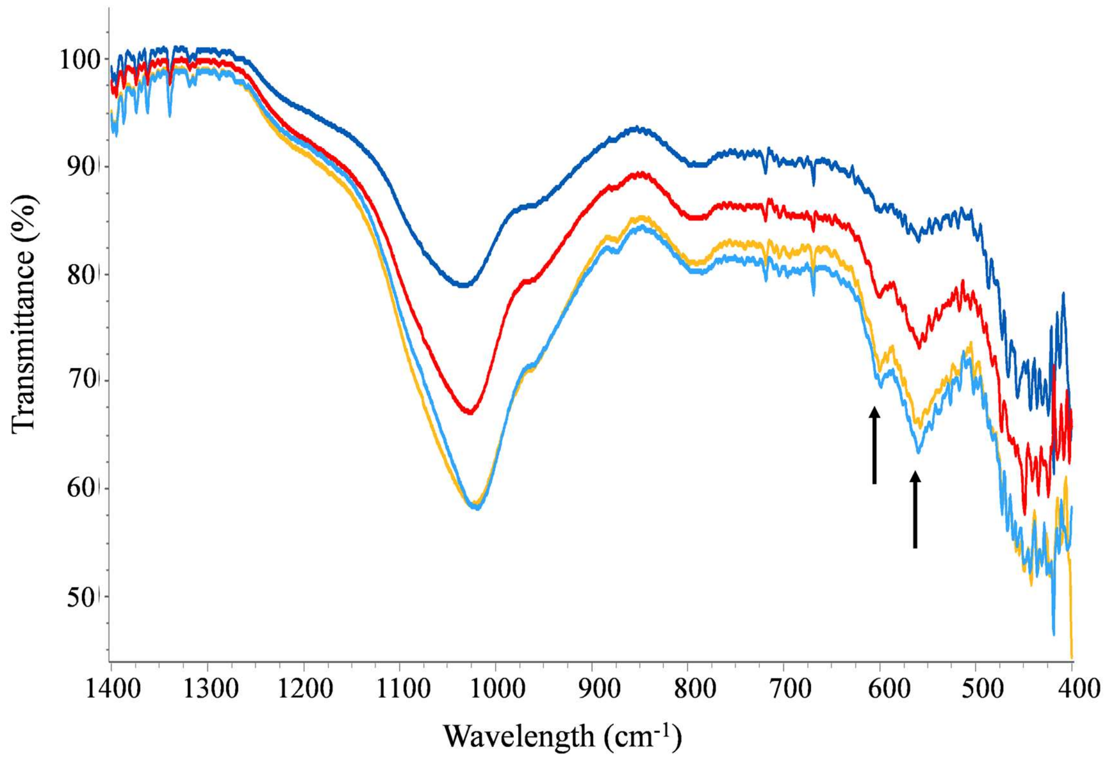

Figure S3. FT-IR spectra of MBG3.6 and MBG5.3 loaded with F after 72 (blue-red) and 168 (orange–light blue) in SBF.
